# Double aneurysm of the superior mesenteric artery: case report

**DOI:** 10.1590/1677-5449.005818

**Published:** 2018

**Authors:** Cristiano Torres Bortoluzzi, Orli Franzon, Rodrigo Viana, Letícia Mattiello, Anderson Henrique da Silva Stahelin

**Affiliations:** 1 Instituto de Cardiologia de Santa Catarina – ICSC, Departamento de Cirurgia Vascular e Endovascular, São José, SC, Brasil.; 2 Hospital Regional de São José Homero de Miranda Gomes – HRSJHMG, Departamento de Cirurgia Geral, São José, SC, Brasil.

**Keywords:** superior mesenteric artery, aneurysm, dacron graft

## Abstract

Superior mesenteric artery aneurysms are rare, accounting for less than 0.5% of all intra-abdominal aneurysms. They are mainly caused by atherosclerosis and septic emboli resulting from bacterial endocarditis. Although uncommon, these aneurysms are considered dangerous because of possible complications such as rupture with hemorrhage and intestinal ischemia. Since the consequences can be very serious, early diagnosis and treatment are essential to improve outcomes. Although there is no well-defined consensus, recommended treatments include open surgery, endovascular therapy, and watchful waiting with periodic examinations. In this article we report the case of an asymptomatic 58-year-old man with an incidental and unusual finding of two superior mesenteric artery aneurysms. Since anatomy was not favorable for an endovascular approach, open surgery was performed, using a dacron graft to successfully repair the artery.

## INTRODUCTION

 Aneurysms of the superior mesenteric artery (SMA) were first described by Koch in 1951 and are rare, accounting for 5.5 to 8% of cases of visceral aneurysms and less than 0.5% of all intra-abdominal aneurysms. 1 ^,^ 2 They occur in similar proportions of men and women and the majority of patients are in the age range of 45 to 55 years. 1 ^,^ 3 Reported incidence based on autopsy studies is from 1 in 12,000 to 1 in 19,000 cadavers. 3

 In addition to the possibility of hemorrhagic shock in the event of rupture, SMA aneurysms can involve risk of ischemia of the intestine, which must be treated because of its lethal nature. 4

 Modern imaging techniques have improved diagnosis and offer the possibility of safely monitoring the disease. 4 Management is decided on a case-by-case basis, and open surgery, endovascular techniques, and watchful waiting are all possible. Indications in the literature for the most appropriate treatment are controversial, and the decision should be guided by characteristics of the patient and of the aneurysm, including age, comorbidities, aneurysm size, anatomic features, and collateral circulation. 4

 This case report describes a patient with two aneurysms of the SMA, for whom open surgical treatment was chosen because of the size of the aneurysm and anatomy that was not suitable for an endovascular approach. 

## CASE DESCRIPTION

**Figure 1:**
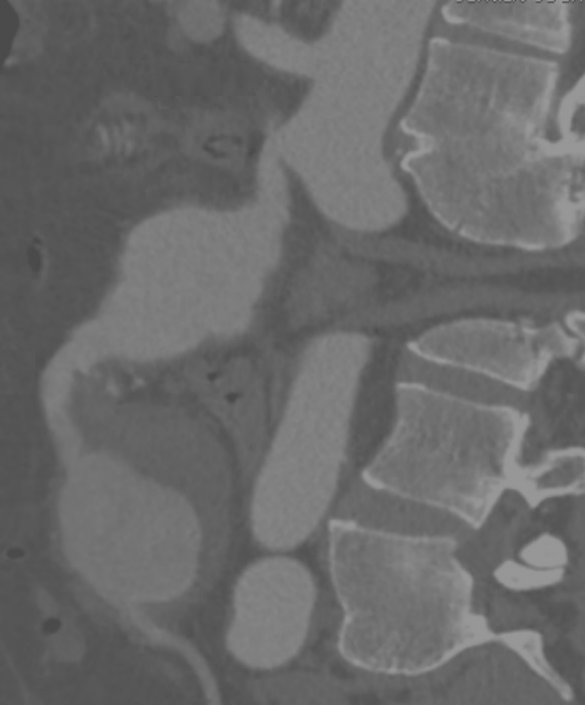
Sagittal angiotomography slice, showing two aneurysms of the superior mesenteric artery.

**Figure 2:**
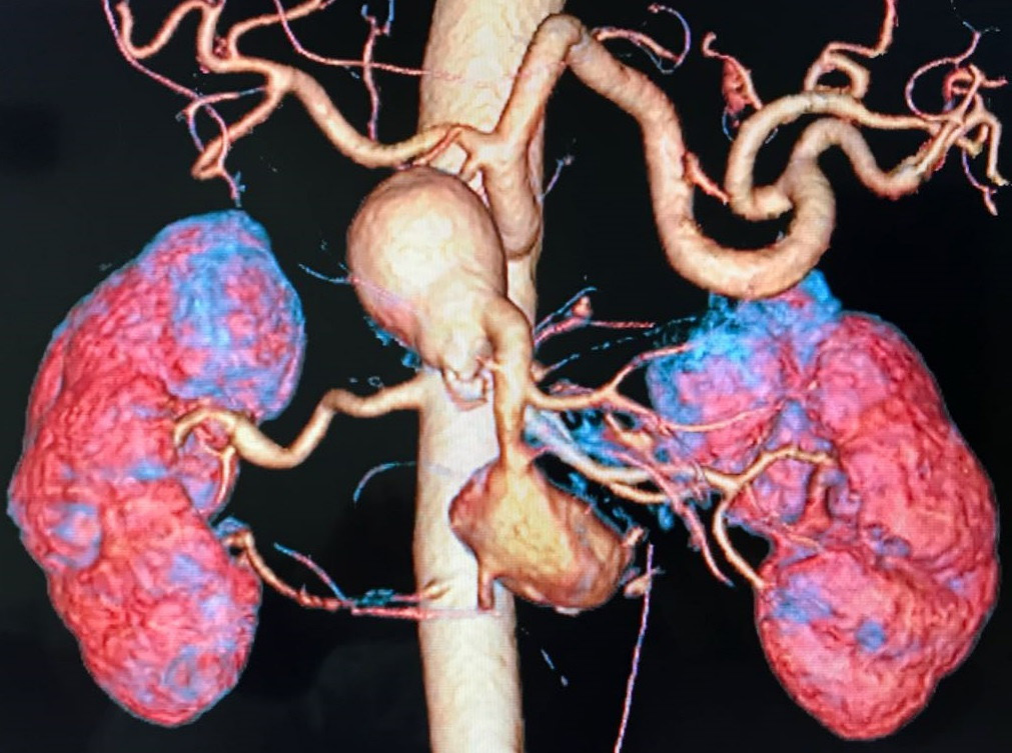
Three-dimensional angiotomography reconstruction, showing two aneurysms of the superior mesenteric artery.

**Figure 3:**
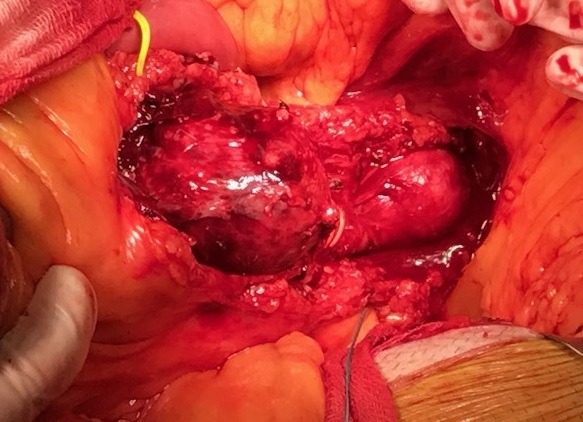
Intraoperative photograph, showing the proximal aneurysm on the left and the distal aneurysm on the right.

**Figure 4:**
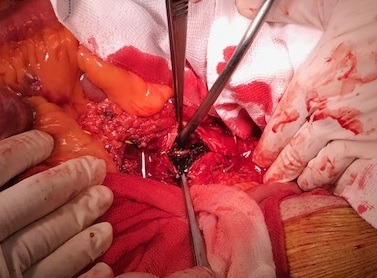
Intraoperative photograph, showing the aneurysm lumen opened to remove the thrombi.

**Figure 5:**
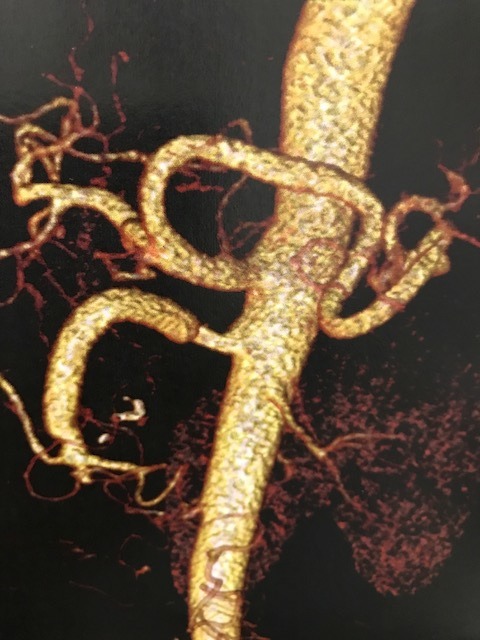
Postoperative control angiotomography, showing the superior mesenteric artery patent, with prosthetic graft.

 The patient was a 58-year-old man, on treatment for hypertension, who was admitted to a vascular surgery service because of an incidental finding of two aneurysms of the SMA, identified during preoperative imaging exams preparatory to repair of an incisional hernia. The patient had no abdominal symptoms and on physical examination his abdomen was flaccid and painless and with a pulsating mobile mass in the epigastrium. Abdominal ultrasonography indicated a partially thrombosed saccular aneurysm in the retroperitoneal space, with no communication with the aorta. Multislice angiotomography revealed two aneurysms of the SMA, a proximal one measuring 5.9 × 5.2 × 5.0 cm and a distal one measuring 5.3 × 3.5 × 3.2 cm ( Figures 1 and 2 ). Since multiple collateral branches emerged from both aneurysm bodies, which meant the endovascular treatment would have involved a risk of damaging the intestinal blood supply, the decision was taken to perform open surgical repair. During the operation, by explorative laparotomy, access to the retroperitoneal space was achieved after performing the Cattell-Braasch maneuver, with medial displacement of the ascending colon and part of the transverse colon, exposing the infrarenal aorta and its branches. This revealed two true aneurysms of the SMA, the larger of which was around 3 cm from the arterial ostium and the smaller approximately 2 cm from the end of the first ( Figure 3 ). It was also possible to observe collateral branches (right colic, ileocolic, jejunal artery, and ileal arteries) projecting from the bodies of these aneurysms. A mesenteric-mesenteric, end-to-end bypass was therefore constructed, using a dacron prosthetic graft, excluding both aneurysms but preserving branches distal of the proximal aneurysm. It was decided to ligate and resect the aneurysms – sending specimens for cultures – and their lumens were opened, revealing large quantities of intraluminal thrombi ( Figure 4 ). Inspection of the abdominal cavity found the intestines to be viable and free from any sign of injury. During the postoperative period, the patient suffered gastrointestinal atony, but responded satisfactorily to prolonged conservative measures. Additionally, on the fifth day after the operation, a control computed tomography revealed signs of hematoma in the hepatorenal recess, managed conservatively to resolution. The patient was discharged from hospital to outpatients follow-up in good clinical condition, 18 days after the operation ( Figure 5 ). There was no bacterial growth in the culture of the aneurysm segment. 

## DISCUSSION

 Aneurysms of the SMA are the third most common type of visceral aneurysm, after aneurysms of the splenic artery and the hepatic artery. In almost all cases, including the case of the patient described here, the aneurysm is within the first 5 proximal cm of the SMA and does not involve the projection of the middle colic artery. 5 ^-^ 7

 Infectious etiologies caused by septic embolism, provoking what are known as mycotic aneurysms, generally affect patients under the age of 50 who have a history of bacterial endocarditis and intravenous drug use. Other causes that merit mention are atherosclerosis (primarily in elderly patients), connective tissue diseases, rheumatoid arthritis, pancreatitis, trauma, Behçet’s disease, neurofibromatosis, and syphilis. 3 ^,^ 8 ^-^ 10 Historically, infectious etiology was considered the principal cause of aneurysms of the SMA, responsible for around 60% of cases. 5 However, more recent studies suggest atherosclerosis is the most common causative factor and just 4.8% of the patients have infectious etiologies. Nevertheless, many authors still consider atherosclerosis to be a secondary process. 7

 In contrast with other aneurysms of the visceral arteries, which in general are asymptomatic, more than 90% of aneurysms of the SMA are symptomatic, manifesting with nonspecific abdominal pains and mimicking gastrointestinal pathologies. 8 ^,^ 11 Fever (in mycotic types), nausea, vomiting, jaundice, and gastrointestinal bleeding can also occur. 2 ^,^ 3 Rarely, a mobile abdominal pulsating mass or an abdominal murmur can be detected during a physical examination. 8

 Around half of all SMA aneurysms are diagnosed in the emergency department because of manifestations of spontaneous rupture, where hypovolemic shock, hemoperitoneum, or acute abdomen are the first manifestations. The mortality rate in these cases is 30 to 90%. 3 ^,^ 8 ^,^ 11 Other possible complications include acute thrombosis or distal embolization with acute mesenteric ischemia, potentially lading to erosion of the adjacent intestine, perforation, and bleeding. 5 ^,^ 6 With relation to these complications, de Troia et al. report a mortality rate of 27% in patients with complicated SMA aneurysms and zero mortality among those with uncomplicated aneurysm. 6

 Aneurysms of the SMA have been diagnosed with increasing frequency over recent years, because of greater accessibility and precision of diagnostic imaging methods. 12

 As in the case described here, diagnosis can be made with abdominal ultrasound, which is a rapid, accessible, and inexpensive tool. 13 However, in order to obtain details that are important for treatment, such as location and dimensions and evidence of rupture and thrombi, in addition to searching for collateral blood supply, multislice angiotomography is recommended and is the gold standard examination in this situation. Significant advances in the imaging software for this examination offer high resolution, three-dimensional images of the abdominal aorta and its branches, which are of great use for planning treatment. 6

 Because of the rarity of SMA aneurysms, there is not yet a definitive consensus on their management, but the options described are watchful waiting, open surgery, and endovascular intervention. 5

 In general, it is recommended that all patients with mycotic SMA aneurysm should undergo aneurysm repair of some type, as should patients with aneurysms larger than 25 mm. People who have aneurysm with sizes less than or equal to 25 mm can be managed with four-monthly imaging exams. 14

 Open surgery is a management option for patients with low surgical risk ratings and, primarily, for those who are hemodynamically unstable and need emergency repair. 15 However, it is a highly invasive procedure with a significant mortality rate of 15%. 5 In general, proximal and distal ligature of the aneurysm is performed, followed by aneurysmectomy and reconstruction of the SMA with saphenous vein or synthetic graft (dacron, for example), the latter of which is only used when there is no evidence of infection. 1 ^,^ 11 It should be remembered that when the aneurysm is located in a distal part of the SMA and there are no findings of ischemic damage to the small intestine, ligature and excision of the aneurysm may be sufficient, because of collateral circulation. However, when the aneurysm is at a more proximal site, at the junction of the SMA with the aorta, or if there is no evidence of collateral circulation, reconstruction of the SMA is mandatory. 7 Additionally, during an open procedure, careful intraoperative assessment of the appearance of the intestine is obligatory and if the bowel is not viable, the segment involved should be resected during the operation. 1 ^,^ 11 ^,^ 13

 As the techniques and devices used have improved, endovascular repair has become an effective and less invasive option for elective treatment of aneurysms of the SMA, particularly in high-risk patients for whom open surgery is contraindicated. 1 ^,^ 3 ^,^ 11 It is worth pointing out that before endovascular intervention, the patient’s anatomy should be carefully evaluated, considering the relationship between collateral branches and the aneurysm, zones for insertion of stents, and vessel tortuosity and caliber, in order to determine whether the procedure is feasible. 16 This method is associated with low short-term morbidity rates, less postoperative pain, fewer operating site complications, shorter length of hospital stay, earlier return to daily activities, and improved quality of life. 15 However, there are also risks inherent to the method, such as iatrogenic dissection and rupture of the vessel, acute thrombosis, thromboembolization and infectious dissemination in cases with mycotic etiology. 5 Other disadvantages also include high reintervention rates, incomplete exclusion of the aneurysm, unknown durability over the long term, and the need for repeated imaging exams. 16

 Currently, the literature reporting results of endovascular interventions in SMA aneurysms still lacks large reviews and is limited to very small cases series, so it is difficult to assess long-term results. 3 ^,^ 11

 Therefore, even after more than 50 years of development of diagnosis and treatment, an SMA aneurysm remains a serious life-threatening condition. Satisfactory results are only achieved with timely diagnosis and treatment. 6 Soon, if the endovascular approach proves viable and safe over the long term, it may become the ideal choice for treatment of SMA aneurysms in selected patients. 12
