# Supplementary material for: Double aneurysm of the superior mesenteric artery: case report
Source: J Vasc Bras. 2018 Oct-Dec;17(4):328–32. doi: 10.1590/1677-5449.005818 (PMC6375273; doi:10.1590/1677-5449.005818)
Supplement: Translation

# Translation: Duplo aneurisma de artéria mesentérica superior: relato de caso

**Authors:** Cristiano Torres Bortoluzzi, Orli Franzon, Rodrigo Viana, Letícia Mattiello, Anderson Henrique da Silva Stahelin

**Affiliations:** 1 Instituto de Cardiologia de Santa Catarina – ICSC, Departamento de Cirurgia Vascular e Endovascular, São José, SC, Brasil.; 2 Hospital Regional de São José Homero de Miranda Gomes – HRSJHMG, Departamento de Cirurgia Geral, São José, SC, Brasil.

**Keywords:** artéria mesentérica superior, aneurisma, prótese de dácron

## Abstract

Os aneurismas de artéria mesentérica superior são raros, representando menos de 0,5% de todos os aneurismas intra-abdominais. São causados principalmente por aterosclerose e por êmbolos sépticos decorrentes de endocardite bacteriana. Apesar de incomuns, são considerados graves, devido a possíveis complicações como ruptura com hemorragia e isquemia intestinal. Com consequências potencialmente devastadoras, o diagnóstico e o tratamento em tempo hábil são essenciais para se otimizarem os resultados. Ainda sem um consenso bem definido, os tratamentos propostos envolvem cirurgia convencional, terapia endovascular e conduta expectante com exames periódicos. Este trabalho relata o caso de um homem de 58 anos, assintomático, com achado incidental e incomum de dois aneurismas de artéria mesentérica superior. Perante uma anatomia desfavorável à abordagem endovascular, foi realizado o tratamento cirúrgico aberto, utilizando-se prótese de dácron na reconstrução arterial, com sucesso.

## INTRODUÇÃO

 Descritos primeiramente em 1951 por Koch, aneurismas de artéria mesentérica superior (AMS) são raros, representando 5,5 a 8% dos casos de aneurismas viscerais e menos de 0,5% de todos os aneurismas intra-abdominais 1 ^,^ 2 . Afetam homens e mulheres em proporção semelhante, com maioria dos pacientes na faixa etária entre 45 e 55 anos 1 ^,^ 3 . A incidência relatada, com base em estudos de autópsia, é de 1 em 12.000 a 1 em 19.000 corpos 3 . 

 Além de choque hemorrágico em caso de ruptura, aneurismas de AMS podem colocar o intestino em risco de isquemia, demandando, pois, atenção devido à sua natureza letal 4 . 

 Técnicas de imagem modernas proporcionaram melhoria no diagnóstico e segurança no acompanhamento da doença 4 . O tratamento é individualizado, sendo possíveis cirurgia aberta, técnica endovascular e conduta expectante. Indicações sobre terapia adequada são controversas na literatura e devem ser orientadas pelas características do paciente e do aneurisma, como idade, comorbidades, tamanho do aneurisma, aspectos anatômicos e circulação colateral 4 . 

 O presente relato de caso apresenta um paciente com aneurisma de AMS em duplicidade, no qual tratamento cirúrgico aberto foi o escolhido, devido ao tamanho do aneurisma e à anatomia desfavorável à abordagem endovascular. 

## DESCRIÇÃO DO CASO

 Homem, 58 anos, hipertenso em tratamento, admitido no Serviço de Cirurgia Vascular devido a achado imagiológico incidental de dois aneurismas em AMS, durante realização de exames pré-operatórios para correção de hérnia incisional. Sem sintomas abdominais, paciente apresentava-se, no exame físico, com abdômen flácido e indolor e com massa pulsátil e móvel em epigástrio. Uma ultrassonografia de abdômen demonstrou um aneurisma sacular parcialmente trombosado em retroperitônio, sem comunicação com a aorta. Já uma angiotomografia *multislice* evidenciou dois aneurismas de AMS, um proximal medindo 5,9 × 5,2 × 5,0 cm e outro distal medindo 5,3 × 3,5 × 3,2 cm ( Figuras 1 e 2 ). Devido à presença de múltiplos ramos colaterais emergindo do corpo de ambos os aneurismas – o que dificultaria a terapêutica endovascular, sob risco de acometimento da irrigação intestinal –, optou-se pela correção cirúrgica aberta. No ato operatório, através de laparotomia exploradora, o acesso ao retroperitônio ocorreu após realização da manobra de Cattell-Braasch, com mobilização medial do cólon ascendente e parte do cólon transverso e exposição da aorta infrarrenal e seus ramos. Notaram-se, assim, os dois aneurismas verdadeiros em AMS, o maior distando cerca de 3 cm do óstio arterial e o menor distando aproximadamente 2 cm do término do primeiro ( Figura 3 ), além de ramos colaterais (artérias cólica direita, ileocólica, jejunais e ileais) projetando-se do corpo desses aneurismas. Realizou-se, então, um *bypass* mesentérico-mesentérico término-terminal com prótese de dácron, excluindo ambos os aneurismas mas preservando ramos distais ao aneurisma proximal. Em seguida, optou-se por ligadura e ressecção dos aneurismas – enviado material para cultura –, além da abertura luminal desses, havendo grande quantidade de trombos intraluminais ( Figura 4 ). Durante inspeção da cavidade abdominal, as alças intestinais demonstraram-se viáveis, sem sinais de qualquer injúria. No período pós-operatário, o paciente apresentou-se com íleo paralítico, havendo resposta satisfatória após medidas conservadoras prolongadas. Além disso, no quinto dia pós-operatório, em tomografia computadorizada de controle, notaram-se sinais de hematoma em espaço hepatorrenal, tratado de forma expectante, com sucesso. O paciente recebeu alta hospitalar em boas condições clínicas, após 18 dias do ato operatório, com seguimento ambulatorial ( Figura 5 ). No exame de cultura do segmento de aneurisma não houve crescimento bacteriano. 

**Figura 1:** Angiotomografia em corte sagital evidenciando duplo aneurisma em artéria mesentérica superior.

**Figura 2:** Angiotomografia com reconstrução tridimensional evidenciando duplo aneurisma em artéria mesentérica superior.

**Figura 3:** Fotografia intraoperatória demonstrando aneurisma proximal, à esquerda, e aneurisma distal, à direita.

**Figura 4:** Fotografia intraoperatória revelando lúmen de aneurisma aberto para a retirada de trombos.

**Figura 5:** Angiotomografia de controle pós-operatório evidenciando perviedade da artéria mesentérica superior, com prótese artificial.

## DISCUSSÃO

 Aneurisma de AMS é o terceiro aneurisma visceral mais comum, atrás apenas dos aneurismas de artéria esplênica e de artéria hepática. Em quase todos os casos, inclusive no paciente relatado neste trabalho, o aneurisma está presente nos 5 cm proximais da AMS, sem envolver a projeção da artéria cólica média 5 ^-^ 7 . 

 Etiologias infecciosas, por embolia séptica, promovendo os chamados aneurismas micóticos, acometem geralmente pacientes abaixo de 50 anos, com histórico de endocardite bacteriana e uso de drogas intravenosas. Outras causas a serem destacadas são aterosclerose (principalmente em pacientes idosos), doenças do tecido conjuntivo, artrite reumatoide, pancreatite, trauma, doença de Behçet, neurofibromatose e sífilis 3 ^,^ 8 ^-^ 10 . Historicamente, a etiologia infecciosa foi considerada a principal causa de aneurisma de AMS, responsável por cerca de 60% dos casos 5 . No entanto, estudos recentes sugerem a aterosclerose como fator causador mais comum, sendo que apenas 4,8% dos pacientes apresentariam etiologia infecciosa. Todavia, muitos autores ainda consideram a aterosclerose como um processo secundário 7 . 

 Ao contrário de outros aneurismas de artérias viscerais, em geral assintomáticos, mais de 90% dos aneurismas de AMS são sintomáticos, manifestando-se como dor abdominal não específica e mimetizando patologias gastrointestinais 8 ^,^ 11 . Febre (nos tipos micóticos), náuseas, vômitos, icterícia e sangramento gastrointestinal também podem ocorrer 2 ^,^ 3 . Raramente, uma massa pulsátil móvel abdominal ou um sopro abdominal podem ser encontrados no exame físico 8 . 

 Cerca de metade dos aneurismas de AMS são diagnosticados no departamento de emergência devido à manifestação de ruptura espontânea, quando choque hipovolêmico, hemoperitônio ou abdome agudo são as primeiras manifestações. A taxa de mortalidade nesses casos é de 30% a 90% 3 ^,^ 8 ^,^ 11 . Outras complicações possíveis incluem trombose aguda ou embolização distal com isquemia mesentérica aguda, levando potencialmente à erosão no intestino adjacente, perfuração e sangramento 5 ^,^ 6 . Diante dessas complicações, de Troia et al. indicam uma taxa de mortalidade de 27% em pacientes com aneurisma de AMS com alguma complicação e ausência de mortalidade naqueles com aneurisma não complicado 6 . 

 Os aneurismas de AMS têm sido diagnosticados com frequência crescente nos últimos anos, devido à maior acessibilidade e acuidade dos métodos diagnósticos de imagem 12 . 

 O diagnóstico, tal como no caso relatado, pode ser alcançado com ultrassom abdominal, uma ferramenta acessível, rápida e barata 13 . No entanto, para se adquirir detalhes importantes para o tratamento, como localização e dimensões, evidências de ruptura e trombos, além da busca por fluxo colateral, é indicada a realização de uma angiotomografia *multislice* – o exame padrão-ouro para essa situação. Para tal exame, avanços significativos nos *softwares* de imagem possibilitam a obtenção de figuras de alta resolução em três dimensões da aorta abdominal e de seus ramos, auxiliando o planejamento terapêutico 6 . 

 Devido à raridade de aneurismas de AMS, ainda não há um consenso definitivo sobre o manejo dessa afecção, sendo descritas as opções de medida expectante, cirurgia aberta e intervenção endovascular 5 . 

 Em geral, recomenda-se que todos os pacientes com aneurisma micótico de AMS tenham de alguma forma o aneurisma reparado, assim como aqueles com aneurisma maior que 25 mm. Já os indivíduos com aneurisma menor ou igual a 25 mm podem ser submetidos a controle periódico com exames de imagem trianuais 14 . 

 Cirurgia aberta é uma opção de manejo para pacientes de baixo risco cirúrgico e, principalmente, para aqueles hemodinamicamente instáveis que requerem reparo de emergência 15 . No entanto, trata-se de procedimento muito invasivo e com taxa de mortalidade significativa de 15% 5 . É realizada, em geral, ligadura proximal e distal do aneurisma, seguida de aneurismectomia e reconstrução da AMS com veia safena ou prótese sintética (de dácron, por exemplo), essa última utilizada somente se não houver indício de infecção 1 ^,^ 11 . Vale lembrar que, quando o aneurisma está localizado em região distal da AMS e não há achados de alteração isquêmica no intestino delgado, ligadura e excisão do aneurisma podem ser suficientes, devido à circulação colateral. Já quando o aneurisma estiver em um ponto mais proximal, na junção da AMS com a aorta, ou se não houver evidência de circulação colateral, a reconstrução da AMS torna-se mandatória 7 . Além disso, na abordagem aberta, a avaliação intraoperatória cuidadosa do aspecto intestinal é obrigatória e, se houver inviabilidade de alça intestinal, o segmento acometido deve ser ressecado no mesmo ato operatório 1 ^,^ 11 ^,^ 13 . 

 Com o aprimoramento das técnicas e dos dispositivos utilizados, o reparo endovascular surge como opção efetiva e menos invasiva no tratamento eletivo de aneurisma de AMS, particularmente em pacientes de alto risco, em que cirurgia aberta não é indicada 1 ^,^ 3 ^,^ 11 . Vale ressaltar que, antes da intervenção endovascular, a anatomia do paciente deve ser criteriosamente julgada, avaliando-se a relação dos ramos colaterais com o aneurisma, as zonas para inserção de stents e a tortuosidade e o calibre do vaso, a fim de verificar a viabilidade do procedimento 16 . Essa abordagem está associada a taxas de morbidade baixas a curto prazo, menos dor pós-operatória, diminuição das complicações do sítio operatório, menor tempo de internação hospitalar, retorno precoce às atividades e melhora da qualidade de vida 15 . Entretanto, há também riscos inerentes a essa modalidade, como dissecção e ruptura iatrogênica do vaso, trombose aguda, tromboembolização e disseminação infecciosa em casos de etiologia micótica 5 . Outras desvantagens ainda incluem taxas altas de reintervenção, exclusão incompleta do aneurisma, durabilidade desconhecida a longo prazo e necessidade de exames de imagem repetidos 16 . 

 Atualmente, a literatura que relata os resultados de intervenção endovascular em aneurisma de AMS ainda é carente de grandes revisões e limitada a séries de casos muito pequenas, sendo difícil avaliar os resultados a longo prazo 3 ^,^ 11 . 

 Logo, mesmo após mais de 50 anos de avanço no diagnóstico e tratamento, o aneurisma de AMS continua sendo uma condição séria que ameaça a vida. Os resultados satisfatórios só são alcançados através do diagnóstico e tratamento oportuno em tempo hábil 6 . Em breve, se a abordagem endovascular provar ser viável e segura a longo prazo, poderá se tornar a escolha ideal para o tratamento de aneurismas de AMS em doentes selecionados 12 .
